# The Interconnection of Carbon Active Addition on Mechanical Properties of Hybrid Agel/Glass Fiber-Reinforced Green Composite

**DOI:** 10.3390/polym15112411

**Published:** 2023-05-23

**Authors:** Muhammad Irfan Nuryanta, Lugas Gada Aryaswara, Rudolf Korsmik, Olga Klimova-Korsmik, Ariyana Dwiputra Nugraha, Seno Darmanto, Muhammad Kusni, Muhammad Akhsin Muflikhun

**Affiliations:** 1 Department of Mechanical and Industrial Engineering, Faculty of Engineering, Gadjah Mada University, Jl. Grafika No. 2, Yogyakarta 55281, Indonesia; 2 Department of Welding and Laser Technologies, Saint-Petersburg State Marine Technical University, Saint Petersburg 190121, Russia; 3 PLN Research Institute, Jl. Duren Tiga Raya No.102, Kec. Pancoran, Jakarta 12760, Indonesia; 4 Department of Mechanical Engineering, Diponegoro University, Jl. Prof. Jacub Rais, Kota Semarang 50275, Indonesia; 5 Department of Aerospace Engineering, Bandung Institute of Technology, Jl. E ITB Jl. Ganesa No.10, Lb. Siliwangi, Kecamatan Coblong, Kota Bandung 40132, Indonesia; 6 Center of Advanced Manufacturing and Structural Engineering (CAMSE), Gadjah Mada University, Jl. Grafika No. 2, Yogyakarta 55281, Indonesia; 7 Center of Energy Studies, Gadjah Mada University, Sekip K-1A Kampus UGM, Yogyakarta 55281, Indonesia

**Keywords:** GFRP, natural fiber, activated carbon, mechanical properties, VARI

## Abstract

Nowadays, the hybridization of natural and glass fiber has promised several advantages as a green composite. Nevertheless, their different characteristics lead to poor mechanical bonding. In this work, agel fiber and glass fiber was used as reinforcements, and activated carbon filler was added to the polymer matrix of a hybrid composite to modify its characteristics and mechanical properties. A tensile and bending test was conducted to evaluate the effect of three different weight percentages of activated carbon filler (1, 2, and 4 wt%). Vacuum-assisted resin infusion was used to manufacture the hybrid composite to obtain the high-quality composite. The results have revealed that adding 1 wt% filler yielded the most optimum result with the highest tensile strength, flexural strength, and elastic modulus, respectively: 112.90 MPa, 85.26 MPa, and 1.80 GPa. A higher weight percentage of activated carbon filler on the composite reduced its mechanical properties. The lowest test value was shown by the composite with 4 wt%. The micrograph observations have proven that the 4 wt% composite formed agglomeration filler that can induce stress concentration and reduce its mechanical performance. Adding 1 wt% filler offered the best dispersion in the matrix, which can enhance better load transfer capability.

## 1. Introduction

The market demand for lightweight, strong, and environmentally friendly materials makes every producer continue to innovate to answer these challenges by using natural products as raw materials for their manufacturing [1,2,3,4,5,6,7,8]. One of the potential natural resources whose utilization still needs to be further developed is the Gebang plant, Latin name *Corypha Utan*. This plant produces fiber from its leaves that is commonly called agel fiber. These fibers are still limited to being used as raw materials for handicrafts such as bags, webbing, hats, furniture, ropes, etc. This natural fiber has the potential to increase its utilization by making it a raw material in the manufacturing of composites so that it has more value to increase income and utilize the local natural product [6,9,10,11,12,13]. Currently, very few studies use agel fibers as composite reinforcement. In its development, many studies have been carried out on natural fibers, which show that natural fibers can not only be used for handicraft products. Natural fibers are expected to replace the role of glass fibers in automotive manufacturing components [14]. Natural fibers can be applied in material engineering as reinforcement in composite materials because they have advantages such as flexibility, tensile strength, and suitable toughness [15]. The implementation of natural fibers can also be combined with synthetic fibers to produce hybrid composites. Hybrid materials are increasingly widespread and offer advantages, especially in the automotive sector [16,17,18,19,20,21,22,23].

Composite is a material whose manufacturing combines two materials with different properties to form a new material with other characteristics [4,24]. In general, composite materials consist of matrices and reinforcements, where fiber is used as reinforcement and matrices are used as a binder. Mixing these two elements will create a material with different chemical and mechanical properties. Mixing the constituent materials to be homogeneous can increase the strength of the material [5,25,26,27]. The use of composite materials with synthetic fiber reinforcement in the form of glass fibers is often found in shipbuilding. The manufacturing of ships with fiberglass-based composites has many reasons, including high resistance to seawater, suitable mechanical properties, and being lightweight [28,29]. The increasing demand for composite materials has resulted in the composite industry in Indonesia having to find other alternatives to replace synthetic fibers, which negatively impact the environment [30]. The development of composites so far still revolves around synthetic fibers that are not renewable because they do not come from agricultural products whose availability is limited.

For this reason, developing composite materials with natural fibers as reinforcement is essential because of their abundant availability and safety for the environment. In addition to these elements being environmentally friendly, natural fibers are also expected to have an affordable price, abundant availability, and quality [31,32]. The use of natural fibers can be an alternative to glass fiber-based structures. In addition, the renewable aspect of natural fibers can be a particular advantage compared to synthetic fibers that require high manufacturing energy [33,34]. However, using natural fibers to strengthen composites has several limitations, including the interaction between the fiber and matrix [34]. The interaction ability between the fiber and matrix is considered to be evaluated. Suitable interaction means suitable load transfer. Effective load transfer can produce a strong and light material, which is the primary purpose of making composites.

The difference in properties between natural fibers and matrices is a challenge. Natural fibers have hydrophilic properties, while commonly used matrices such as polyester and epoxy are hydrophobic [35]. These property differences make it challenging to create suitable interactions, leading to poor interface bonding. To overcome this problem, specific treatments to improve fiber and matrix interactions need to be carried out [36]. One of the chemical treatments commonly used is alkalization. Alkali treatment attempts to modify the fiber surface to create a suitable surface bond between the matrix and the fiber [37]. Three solutions can be used as alkalization methods: KOH, LiOH, and NaOH. NaOH solution is a solution that is often used to modify natural fibers [32,37,38,39,40]. The content of lignin and cellulose can interfere with the fiber bonding process with the matrix. Fibers must be soaked with NaOH to remove the wax and lignin layer [41]. In addition, alkaline NaOH treatment with a specific presentation and time can increase the roughness on the fiber’s surface, resulting in better fiber and matrix interactions [39,40].

Research shows that in hybrid composites, the order of the laminate arrangement of a composite also affects the mechanical properties, especially the flexural strength [42]. A suitable manufacturing process and knowing the proper order of laminates can improve the mechanical properties of hybrid composites even with the same proportion of reinforcement. The manufacturing of composites with a hybrid process is an effort to obtain better mechanical strength [43]. Some factors affect the mechanical aspects of [11,44,45,46,47,48,49]. Woven fiber composites can be varied in several ways, such as variations in the arrangement, the number of layers, and the orientation of the fibers. These variations also produce different mechanical strengths [50]. The combination of reinforcement with different geometries and elements aims to obtain better mechanical properties and improve its function [51]. Thermoset-based composites can be reasonably susceptible to internal damage, such as cracks and voids, creating durability problems. One solution to this problem is the use of fillers. Adding filler to the composite can improve the composite’s mechanical, physical, and other properties [52] and thermal stability and flame retardancy [53]. Many filler materials are used to reinforce composite laminates. One of the fillers that were used and investigated by many researchers was activated carbon (AC). AC can be defined as a class of amorphous carbonaceous materials characterized by large porosity and internal surface area [54,55,56]. Nawras et al. [57] investigated the addition of activated carbon filler with variations of 1%, 3%, 5%, and 10%. The matrix used is polyester with hemp fiber reinforcement treated with NaOH. The tensile test results showed an increase in the composite’s tensile strength and bending strength with the addition of 3% filler. Activated carbon filler prevents slip on the polyester chain, and this causes an increase in the tensile modulus of the composite. An increase in filler concentration of more than 3% resulted in a decrease in the tensile modulus. The effect of adding filler to the composite was also investigated by Sravanthi et al. [58] by performing impact testing on GFRP with added micro carbon filler. Bidirectional glass fiber is arranged in 5 layers with 5, 10, and 15 wt% filler variations with a hand lay-up manufacturing process. The test results show that adding 5 wt% filler produces the best strength compared to other percentages. Adding filler can also improve thermal stability and flame retardancy [59]. 

Singh et al. [60] investigated the effect of adding fishbone filler in flax and carbon fiber-reinforced composites. The concentration of filler addition is 0%, 2%, and 5% in each arrangement of the lamina. The tensile strength of the composite increased with the addition of fishbone filler and carbon fiber, but the strength decreased when flax fiber was added. The test specimen obtained the highest tensile strength with a concentration of 30% carbon fiber, 0% hemp fiber, and 5% filler. The failure of the specimen in the test occurs due to the agglomeration of filler particles. This can trigger voids and cracks in the composite. Uneven distribution of filler is a challenge. Therefore, it is necessary to add the optimal percentage of filler so that uniformity in filler distribution can be achieved. Research on activated carbon filler from coconut shells was also carried out by Sari et al. [61]. The research used corn frond fiber and coconut shell-activated carbon filler with 5% and 10% variations. The natural fibers used are not treated first. The filler mixing process is also performed manually. The influence of the uneven distribution of fibers and fillers in the presence of voids causes the strength of the composite to be not optimal.

Several studies about carbon filler and natural fiber have been conducted in the previous work but still less discussed about agel fiber. The agel fiber is a promising natural fiber from the Gebang plant with several advantages. Hence, combining activated carbon filler and a hybrid composite consisting of agel fiber will result in the unique characteristics of a hybrid composite. Activated carbon filler has a honeycomb-like shape. Its surface has pores that can increase the contact surface area with the polymer, which will create a strong bond between the filler and the matrix. The use of activated carbon filler can inhibit crack propagation, which will increase the toughness of the composite. This study focuses on adding activated carbon filler to the strength of the composites reinforced with agel and glass fibers. The novelty in this study is using a variation of activated carbon filler of 1%, 2%, and 4% combined with agel and glass fiber reinforcement. Agel fibers are treated first, and the mixing process is controlled by the speed and time of stirring. The composite manufacturing process uses the vacuum-assisted resin infusion method, which is expected to increase the strength of the composite because it can minimize the occurrence of voids.

## 2. Materials and Methods

### 2.1. Materials

**Figure 1:**
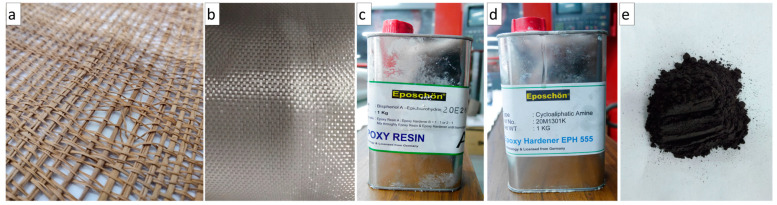
Shows materials used in this study. (**a**) Agel fiber; (**b**) woven glass fiber; (**c**) epoxy resin; (**d**) Epoxy Hardener EPH 555; (**e**) filler activated carbon.

The manufacturing of composites in this study utilizes natural fibers and synthetic fibers. The natural fiber used is an agel fiber purchased at a craft shop in Yogyakarta. Glass fiber was expected to improve the properties of the composite. Figure 1 shows the fiber and matrix used in this study. The present study used two different fibers (agel and glass). The agel fibers are shown in Figure 1a, and the glass fiber used is a woven type with a weight of 200 gsm, shown in Figure 1b. The matrix that was used was Epoxy EPR 174, which consists of bisphenol A (see Figure 1c), while Epoxy Hardener EPH 555, which consists of cycloaliphatic amine (Figure 1d). For the filler materials, coconut shell-activated carbon filler with 1000 mesh sizes is used (Figure 1e).

### 2.2. Methods

#### 2.2.1. Mixing Epoxy Resin and Filler

**Figure 2:**
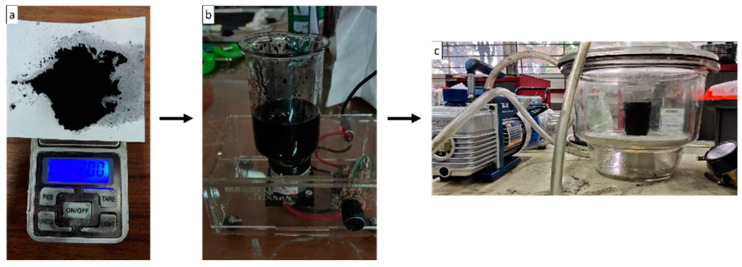
Preparation process. (**a**) Weighing filler, (**b**) stirring filler and matrix, and (**c**) degassing.

The effect of filler concentration was investigated in this study. Coconut shell-activated carbon filler was used. Adding filler to the composite is expected to increase its mechanical strength. It is necessary to conduct a study related to the percentage of filler content to analyze the effect of adding filler to produce maximum composite strength. The filler preparation process begins with weighing the filler, mixing the resin and filler using a magnetic stirrer, and the degassing process, which can be seen in Figure 2. Each variation of the specimen contains a different concentration of filler, namely 0%, 1%, 2%, and 4% of the weight of the matrix.

#### 2.2.2. Manufacturing Composite

**Figure 3:**
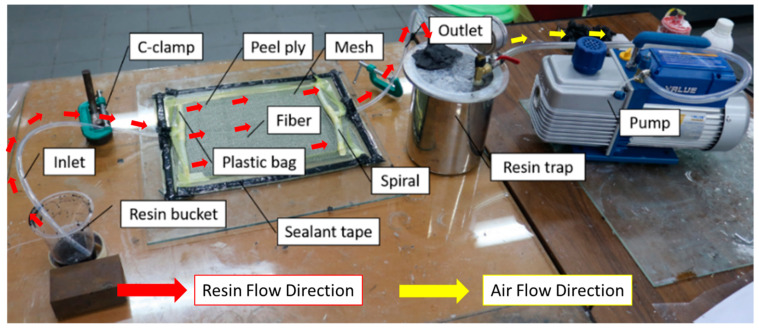
Experimental set-up of vacuum-assisted resin infusion. Red arrow for resin flow, and yellow arrow for air flow.

**Figure 4:**
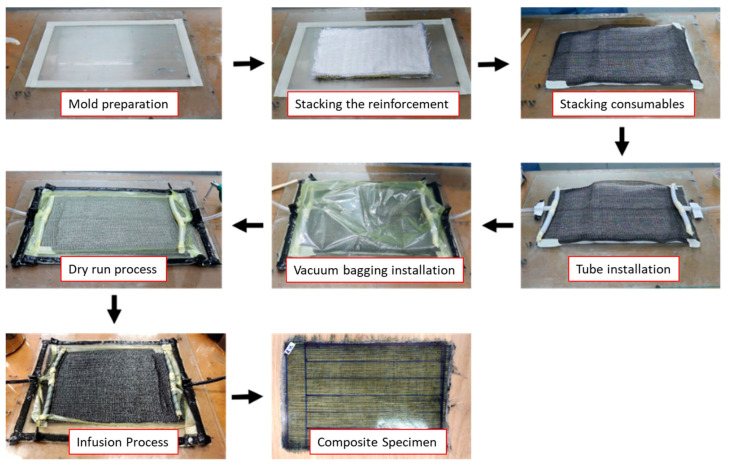
Vacuum-assisted resin infusion process.

**Figure 5:**
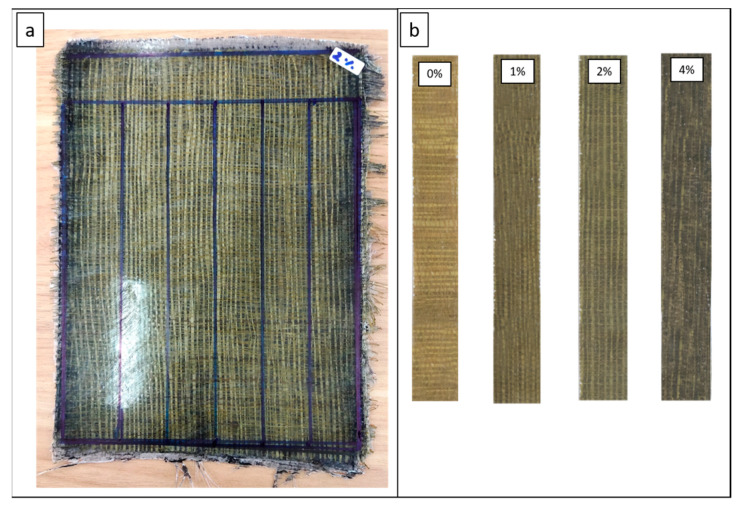
(**a**) Composite plate, (**b**) fiber distribution in each variation.

**Figure 6:**
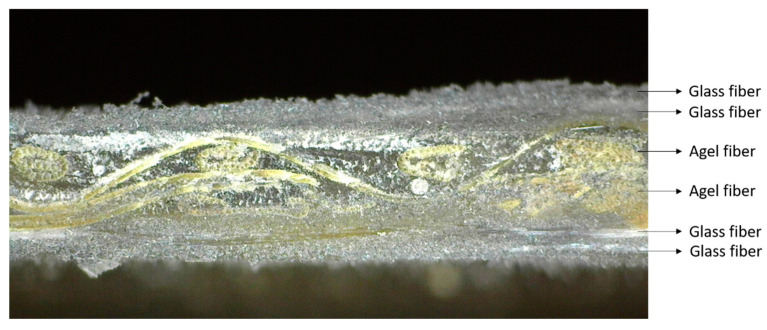
Stacking sequence composite laminate.

Vacuum-assisted resin infusion was used in the present study to manufacture hybrid composite laminates. This method has its challenges because the equipment preparation process is more complex than the hand lay-up method. More tools and materials make this method require a longer manufacturing preparation and higher costs. The resin contained in the plastic container is flowed into the mold cavity by the sucked of a vacuum pump. Before resin flow, the impression cavity containing agel fibers and glass fibers was ensured in a vacuum or without air. The vacuum condition in the mold cavity allows the resin to flow into the fiber without being blocked by air. This manufacturing method can produce composite materials that are better in strength because it can minimize the voids that often occur in other methods, such as hand lay-up. The experimental set-up and composite manufacturing process using the vacuum-assisted resin infusion method can be seen in Figure 3 where the resin flow direction are illuastrated in red arrow and the air flow direction are illustrated with yellow color. In the manufacturing process of the laminates, the detailed process can be seen in Figure 4. Fiber distribution in the composite specimen and the stacking sequence composite used in this study can be seen in Figure 5 and Figure 6.

**Figure 7:**
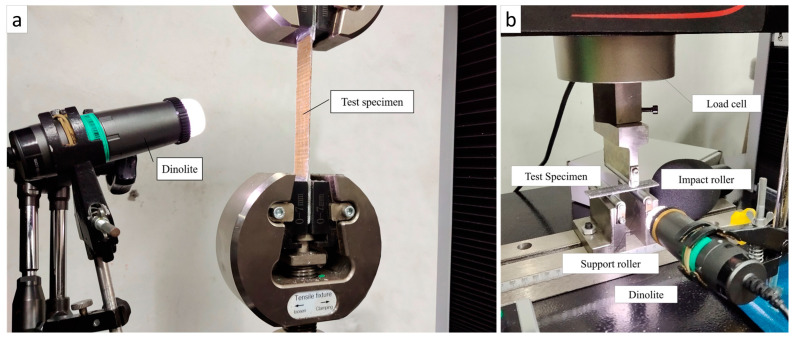
Tool set-up on (**a**) tensile test, (**b**) flexural test.

Mechanical tests carried out in this study are tensile testing and bending testing. Dimensional and testing standards refer to ASTM D3039 for tensile tests and ASTM D790 for bending tests. The process of cutting the composite plate into a test specimen is carried out using the help of a composite cutting tool. Tensile and flexural testing was performed with the Carson UTM CRN-50 model. Prior to testing, all test specimens were measured for dimensions. The testing process on tensile and flexural loading can be seen in Figure 7.

## 3. Results and Discussion

### 3.1. Volume Fraction

**Figure 8:**
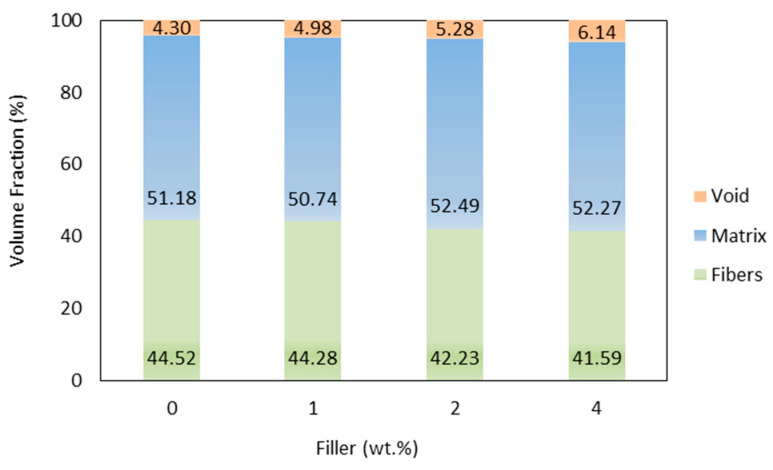
Fiber, matrix, and void volume fractions comparison between the parameters combinations.

The fiber volume fraction, matrix, and percentage of voids contained in the specimen for each filler concentration are shown in Figure 8. The strength of the composite material depends on the number of fibers and the matrix content indicated by the volume fraction of the fiber and matrix. Voids can be a stress concentration in the composite. The presence of voids is one factor affecting the strength of the composite material. In this study, the presence of voids is obtained by calculating the difference between the theoretical and experimental density of the test object and then dividing it by the theoretical density.

The highest fiber volume fraction was obtained by specimens without filler, with 44.52%, while the lowest was 4% filler, with 41.59%. The manufacturing method used is very influential on the volume fraction of the fiber in the composite. The vacuum-assisted resin infusion method’s advantage was shown in the specimen laminates, where it can produce composites with a suitable fiber volume fraction. This laminate is thinner but has better load resistance than conventional methods such as hand lay-up. Composites with a filler content of 4% have the highest percentage of voids than other variations. The findings confirm that an increase in the number of fillers increases the volume of voids, which will affect the performance of the composite. The addition of gel fibers to the composite plays a role in reducing the fiber’s volume fraction. Natural fibers that are woven will create space between the fibers. Moreover, the space between the laminae becomes a cavity the matrix will fill. As a result, the specimens have a fiber matrix ratio with a low fiber content [12].

### 3.2. Dispersion Analysis

**Figure 9:**
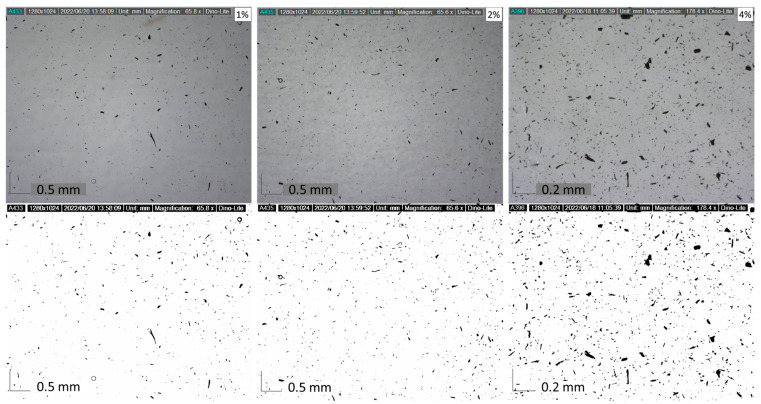
Filler distribution of various specimens.

Dispersion, shape, and concentration of filler in each variation affect the strength of the composite. The filler dispersion phenomenon can be observed using the image and video output carried out with a Dinolite digital microscope. The observed filler dispersion phenomenon can be used as a reference for the strength of the specimen. Observations also include phenomena that occur in the specimen. Figure 9 shows the shape and distribution of filler in the matrix for each variation. The non-uniform filler will affect the strength of the composite. Figure 8c shows the filler dispersion of 4%, more dense than the 1% and 2% filler. The agglomeration phenomenon has been found in the 4% filler. The several big black dots are marked as an agglomeration of 4% activated carbon filler in the matrix. The dispersion of 1% and 2% filler is more uniform, marked by black dots (activated carbon filler) spread evenly in the matrix. Hence, adding 1% or 2% filler can reduce the chance of the agglomeration phenomenon formed by activated carbon filler.

**Figure 10:**
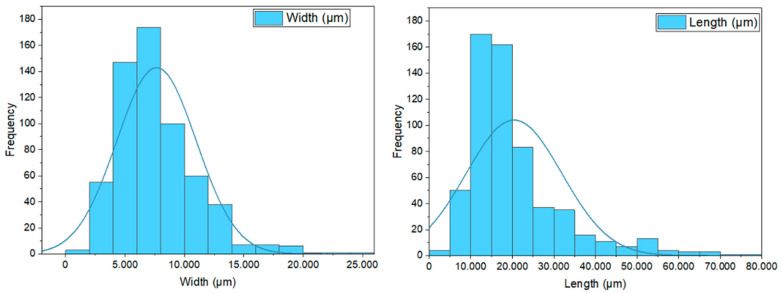
Activated carbon filler size.

The size of the activated carbon filler used can be seen in Figure 10. The aspect ratio of the filler’s length (L) and diameter (D) affects the composite’s strength. The higher the aspect ratio (L/D) increases, the strength of the composite. The size of the activated carbon filler that has been observed has a width ranging from 1.4 µm to 25.1 µm, with an average width of 7.6 µm. Meanwhile, the filler length ranges from 3.8 µm to 76.4 µm, with an average length of 20.3 µm. A smaller filler size will result in a large filler surface area in contact with the resin. A wider surface area will increase the composite’s strength due to more efficient stress transfer.

### 3.3. Tensile Test

**Figure 11:**
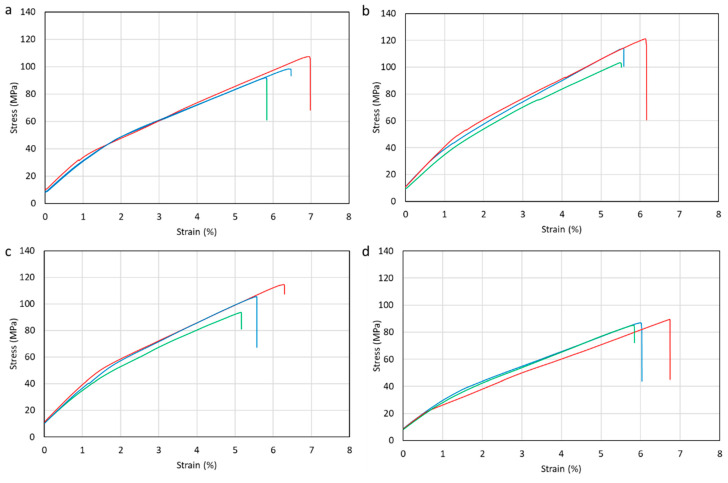
Stress–strain curve of (**a**) 0%, (**b**) 1%, (**c**) 2%, (**d**) 4% filler. Red line is sample 1, blue line is sample 2, and green line is sample 3.

**Figure 12:**
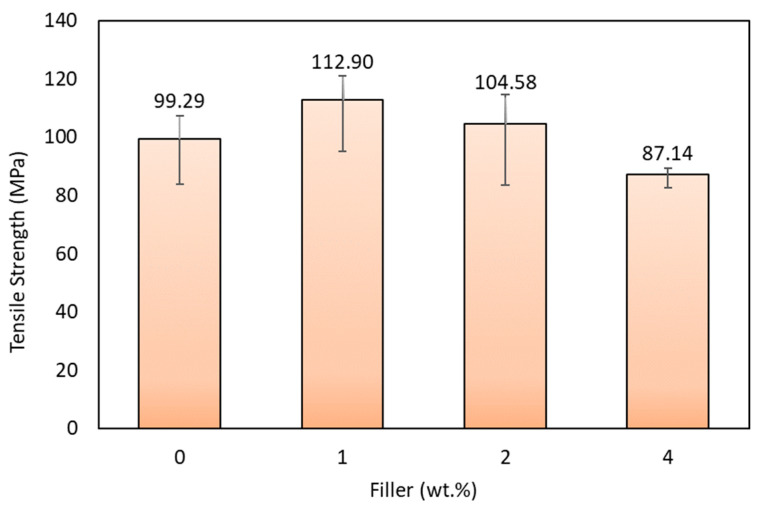
Tensile strength of various specimens.

The stress–strain value from the present study is shown in Figure 11. The value of the stress–strain graph uses a pre-load of 400 N in all variations. The tensile test results showed that adding coconut shell-activated carbon filler affected the tensile strength of the glass fiber composite and agel fiber. The increase in tensile strength was obtained by composites with 1% and 2% fillers, while specimens with a filler content of 4% experienced a decrease in tensile strength compared to specimens without fillers. The results showed that 1% filler has the highest tensile strength with 112.90 MPa, while the lowest was a composite with 4% filler with 87.14 MPa. Figure 12 shows the average tensile strength results in a graph.

From the graph, adding 1% of filler can improve the mechanical properties of the composite. This is because the filler powders in the matrix create a suitable surface bond between the fiber and the matrix, which causes the load transfer capacity between the activated carbon filler and the matrix interaction. Composites with a filler content of 1% experienced an increase in tensile strength of 13.7% than composites without fillers. In contrast, the composite with the addition of 4% filler gets the lowest tensile strength value. The weak bond between fiber and matrix due to agglomeration and adding more filler causes decreased tensile strength in composite specimens with a filler content of 4%. This confirms that filler agglomeration is a crucial factor that causes poor properties of composite materials. Void and filler aggregation in the matrix caused stress concentration [62].

**Figure 13:**
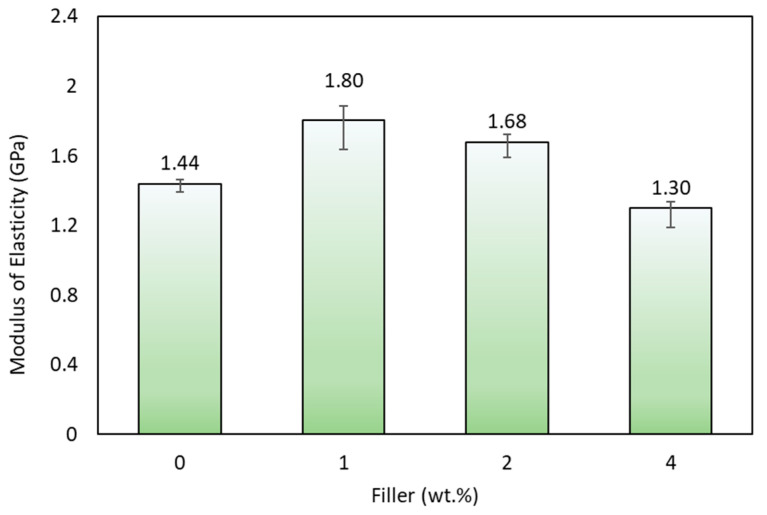
Modulus elasticity of various specimens.

The uniform filler distribution in composite specimens with a filler concentration of 1% makes the load given during the testing process uniformly carried. The epoxy resin wets the fiber well, creating a strong adhesion between the fiber and the matrix. In contrast, the addition of 4% filler makes a non-uniform distribution of the filler. It is caused by the increase in the viscosity of the matrix as the filler concentration increases, which can reduce the flow rate of the matrix when wetting the fiber. The increase in tensile strength was followed by increased stiffness in the specimen. The results illustrated in Figure 13 shows that the tensile modulus with a filler content of 1% obtained the highest tensile modulus of elasticity, followed by specimens with 2% and 0% content. In composites with a filler content of 1%, there was an increase in tensile modulus of elasticity by 25% compared to composites without fillers. In comparison, specimens with a filler content of 4% have the lowest tensile modulus of elasticity. The previous results [63] showed that increasing the number of fillers will increase the strength and stiffness of the composite material.

The increase in strength and modulus is related to the filler’s shape and size, which allows suitable interaction with the epoxy resin, thereby inhibiting the mobility of the epoxy chain. The irregular shape of the coconut shell-activated carbon filler makes the values obtained not as suitable as composites with fillers with a uniform shape. The decreased composite performance (strength) was related to the weak fiber and matrix interaction. The filler’s dispersion and shapes are not uniform. Followed by the voids in the composite can also decrease the composite’s modulus of elasticity [64].

### 3.4. Flexural Test

**Figure 14:**
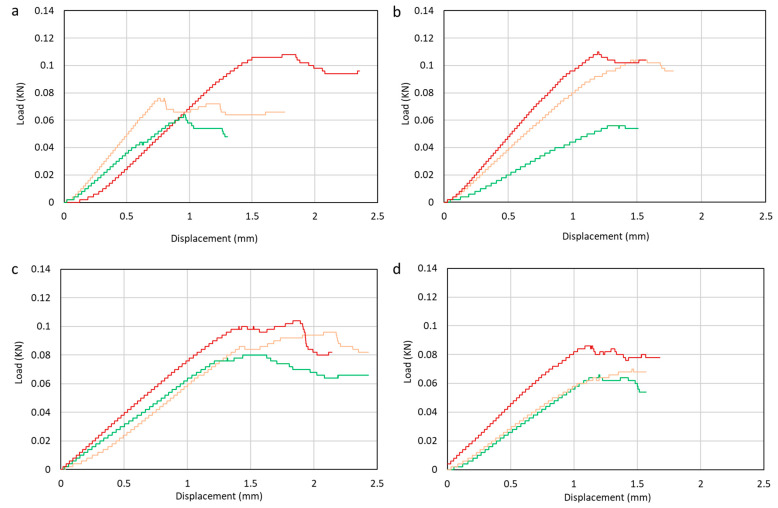
Load–displacement curve of (**a**) 0%, (**b**) 1%, (**c**) 2%, (**d**) 4% filler. Red is sample 1, green sample 2, and orange sample 3.

**Figure 15:**
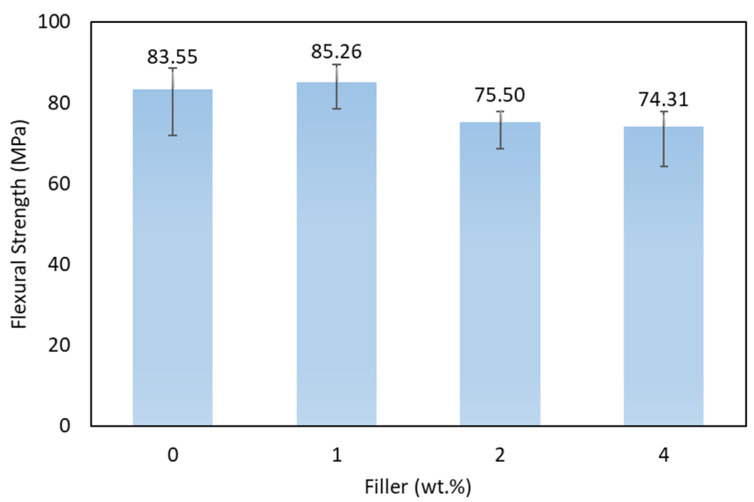
Flexural strength of various specimens.

The flexural test is carried out using UTM following ASTM D790. After testing, the test results are in the form of a load–displacement graph shown in Figure 14. From the results of the flexural tests carried out, the data obtained in the form of the average flexural strength of each variation of filler concentration can be seen in Figure 15.

The bending strength graph shows the highest strength obtained by the composite with 1% filler, which is 85.26 MPa, and the lowest is composite with 4% filler content, which is X74.31 MPa. Activated carbon filler increased the specimen’s strength with a suitable adhesive between the filler and the matrix at 1% filler concentration. The optimum filler percentage can establish a uniform distribution into the composite. This creates a suitable bond chain that can withstand the maximum load. Increasing the amount of filler can also cause a decrease in bending strength due to agglomeration and non-uniform filler dispersion. The imperfection bonding between matrix and fiber can cause poor adhesion between the matrix and fiber. Poor adhesion can create cavities and become stress concentrations in the composite. It is shown from the results that the filler added to the hybrid composite of glass fiber and agel fiber can increase the bending strength. This is related to the dispersion and adhesion between the fiber and the matrix. This phenomenon is caused by the lack of epoxy matrix chain mobility due to bending loads. The decrease in bending strength is related to filler addition that blended with the matrix. Since the filler is not perfectly dispersed in the matrix, it causes agglomeration. This tent generated micro-cracks that caused the failure of the laminates. The bending strength obtained in the specimen with the addition of 2% filler has decreased strength compared with the specimen without filler. 

The results from the flexural test showed that the manufacturing process of the laminates greatly affected the laminate performance. The distribution of filler is crucial in the manufacturing process. By using vacuum-assisted resin infusion, it can minimize activated carbon filler not spreading well because it is filtered by the fibers used. The lack of distribution of filler caused different composite strengths even in the same composite plate and may offer preliminary failure.

### 3.5. Micrograph Analysis

**Figure 16:**
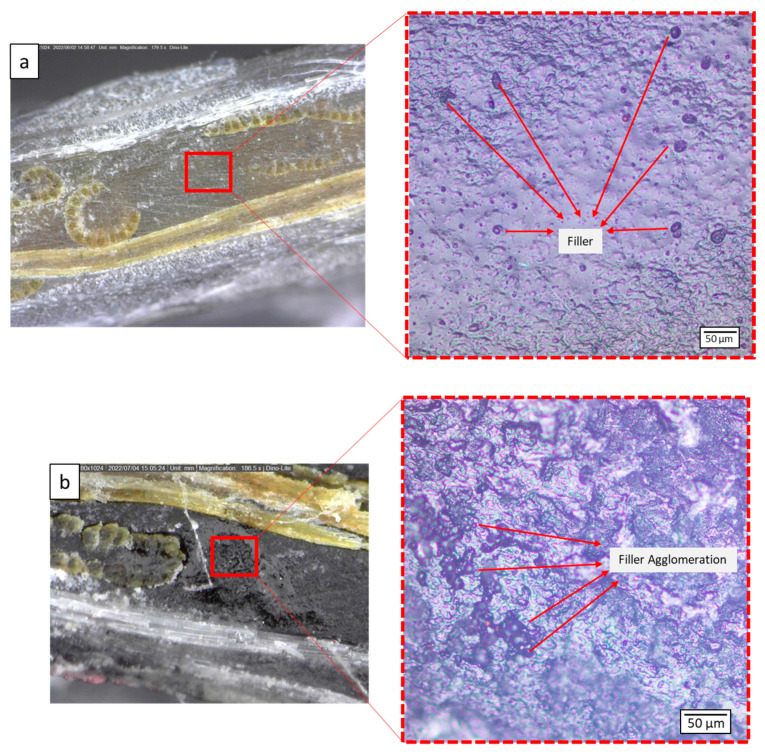
Filler dispertion at (**a**) 1%, (**b**) 4% concentration.

**Figure 17:**
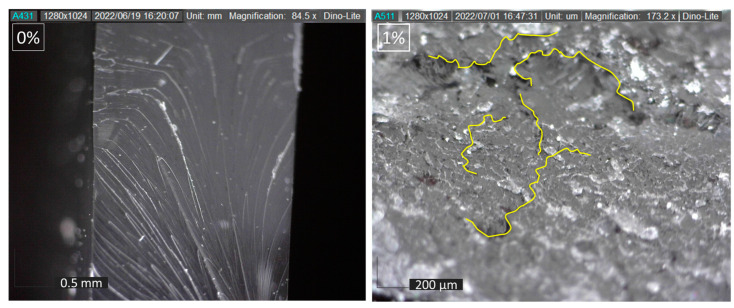
Crack propagation mechanism.

Micrograph analysis has been conducted on the failure specimen shown in Figure 16. The agglomeration phenomenon is due to filler agglomerate and creates a stress concentration around the edges. It can initiate cracks, as shown at 4% filler specimen. This confirms that adding the optimum amount of filler can increase the strength of the composite, but too much filler can create a weakness in the laminates due to agglomeration. The phenomenon of crack propagation that occurs after the tensile test can be seen in Figure 17. Fillers are capable of delaying crack initiation by crack propagation mechanisms such as deflection and blocking the crack bridging.

**Figure 18:**
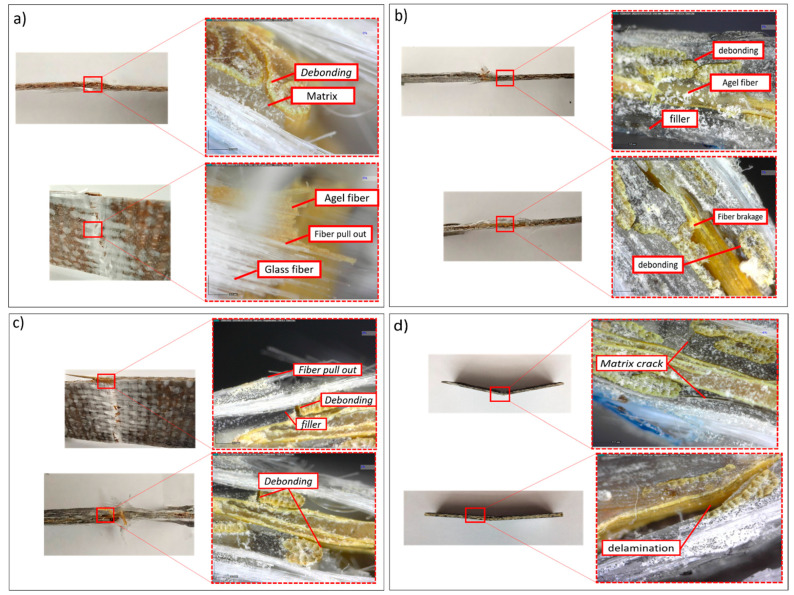
Composite failure mechanism with filler (**a**) 0%, (**b**) 1%, (**c**) 2%, (**d**) 4% filler.

Observations were also made on the fracture cross-section in each variation. Evaluation of the specimens after testing was carried out, as shown in Figure 18. The failure phenomenon in the present study can be used as a reference for the strength of the composite adhesive between fiber and matrix. This is s due to the influence of the filler, which becomes the focus of this micrograph observation.

A hybrid composite consisting of glass and agel fiber with the addition of activated carbon filler was observed. Observations were performed to detect the failure phenomena, including fiber and resin interactions, fiber pull-outs, and other types of failure. Adding filler to the matrix will increase the laminate’s viscosity. Resin with low viscosity will interact easier with the fiber because of the high molecular mobility resulting in a strong bond [65]. In all variations, identical failure phenomena were found. Imperfection bonding between fiber and matrix resulted in debonding and delamination. The filler content in the matrix also affects the fiber-wetting process. Debonding and delamination failure indicates weak matrix adhesion to the fiber. These are due to agglomeration and unperfected fiber wetting by the matrix. Specimens observation showed optimal load transfer capability with a filler content of 1%. This is because the matrix, fiber, and filler can withstand the load optimally. Failure due to the crack matrix found in the specimen with a filler content of 4% indicates that the load transfer process is not optimal.

## 4. Conclusions

Research has been conducted on the effect of activated carbon filler on the mechanical properties of hybrid composites reinforced with agel fibers and glass fiber. From the research results, we obtained the following conclusions:

- The effect of adding filler on tensile strength shows that composites obtain the highest tensile strength by adding 1% filler, which is 112.90 MPa. Composites receive the lowest tensile strength by adding 4% filler, 87.14 MPa. Composites obtained the highest tensile modulus by adding 1% filler was 1.80 GPa, while the lowest was 1.30 GPa in composites with 4% filler;
- The flexural test has revealed that the composite with 1% filler has the highest strength of 85.26 MPa, while the composite with 4% filler shows the lowest bending strength of 74.31 MPa;
- The poor dispersion of 4% filler leads to inducing agglomeration phenomenon. The agglomeration phenomenon reduces the mechanical performance of the hybrid composite. The better dispersion has obtained by 1% filler. The optimum value of filler can enhance the mechanical performance of the composite. The filler’s shape and size allow suitable interaction with the epoxy resin, thereby inhibiting the mobility of the epoxy chain. Suitable interaction confirms that the increase in strength and modulus is related to the optimum value of filler addition;
- Micrograph observations have proven several failures in each test specimen, including debonding, fiber pull-out, and cracks in the fiber and matrix.

Based on the study, adding 1% of activated carbon filler has better mechanical properties. Evaluating the fluidity of the matrix after adding the filler and wetting performance during the vacuum-assisted resin infusion is recommended for further research. The fatigue and water resistance of a bio-filler or a hybrid composite of natural fiber should be evaluated due to hydrophilic characteristics.

## Data Availability

The data can be available upon request to the authors.
